# Performance of a quantitative fecal immunochemical test for detecting advanced colorectal neoplasia: a prospective cohort study

**DOI:** 10.1186/s12885-018-4402-x

**Published:** 2018-05-02

**Authors:** Elizabeth G. Liles, Nancy Perrin, Ana G. Rosales, David H. Smith, Adrianne C. Feldstein, David M. Mosen, Theodore R. Levin

**Affiliations:** 1 Kaiser Permanente Center for Health Research, 3800 N Interstate Ave, Portland, OR 97227 USA; 2 Johns Hopkins School of Nursing, 525 N Wolfe St, Baltimore, MD 21205 USA; 3 Kaiser Permanente Medical Center, 1425 S. Main Street, Walnut Creek, CA 94596 USA

**Keywords:** Colorectal neoplasms, Mass screening, Occult blood, Sensitivity and specificity

## Abstract

**Background:**

The fecal immunochemical test (FIT) is easier to use and more sensitive than the guaiac fecal occult blood test, but it is unclear how to optimize FIT performance. We compared the sensitivity and specificity for detecting advanced colorectal neoplasia between single-sample (1-FIT) and two-sample (2-FIT) FIT protocols at a range of hemoglobin concentration cutoffs for a positive test.

**Methods:**

We recruited 2,761 average-risk men and women ages 49-75 referred for colonoscopy within a large nonprofit, group-model health maintenance organization (HMO), and asked them to complete two separate single-sample FITs. We generated receiver-operating characteristic (ROC) curves to compare sensitivity and specificity estimates for 1-FIT and 2-FIT protocols among those who completed both FIT kits and colonoscopy. We similarly compared sensitivity and specificity between hemoglobin concentration cutoffs for a single-sample FIT.

**Results:**

Differences in sensitivity and specificity between the 1-FIT and 2-FIT protocols were not statistically significant at any of the pre-specified hemoglobin concentration cutoffs (10, 15, 20, 25, and 30 μg/g). There was a significant difference in test performance of the one-sample FIT between 50 ng/ml (10 μg/g) and each of the higher pre-specified cutoffs. Disease prevalence was low.

**Conclusions:**

A two-sample FIT is not superior to a one-sample FIT in detection of advanced adenomas; the one-sample FIT at a hemoglobin concentration cutoff of 50 ng/ml (10 μg/g) is significantly more sensitive for advanced adenomas than at higher cutoffs. These findings apply to a population of younger, average-risk patients in a U.S. integrated care system with high rates of prior screening.

## Background

Colorectal cancer (CRC) is the third-leading cause of cancer-related deaths in the United States, affecting men and women almost equally [1]. Population screening for CRC has reduced disease-related mortality due to high prevalence of resectable precancerous lesions that have a slow progression to clinically invasive cancer [2–6]. The fecal immunochemical test (FIT) has higher patient adherence [7–10] and better sensitivity than guaiac fecal occult blood testing (gFOBT) [11], leading to recommendations for its use in colorectal cancer screening [12–14]. Important questions about the optimal use of this test remain. In particular, it is unclear whether one or two samples should be collected and what hemoglobin cutoff for a positive test would optimize sensitivity and specificity for detection of cancers and advanced adenomas [15, 16]. Organized CRC programs in different parts of the world use fecal tests requiring 1, 2 or 3 fecal samples worldwide, without consensus about the optimal approach [17]. Theoretically, an extra fecal sample may provide the chance to detect an intermittently bleeding, clinically significant neoplasm. However, only a paucity of observational evidence, and no randomized trials address this question.

There are several different types of FIT available [12, 15]. Large trials comparing the effectiveness of colonoscopy with FIT use the OC-Auto-FIT (prior names: OC Micro, OC Sensor) [18, 19], a quantitative FIT with results processed by an automated analyzer. This FIT has been found to be 80% sensitive for cancer when used at the manufacturer-recommended hemoglobin concentration cutoff of 100 ng/ml of buffer, or 20 μg/g of stool [19, 20]. The sensitivity of the OC-Auto FIT can be increased further by lowering the cutoff of hemoglobin concentration for a positive test result [15], although it has been unknown what the trade-off in false positives would be at a lower cutoff if used in an average-risk screening population.

In the Maximizing Yield of the Fecal Immunochemical Test for Colorectal Cancer Screening (MY-FIT) study, we sought to determine the optimal combination of hemoglobin concentration cutoff and fecal samples for the OC-Auto FIT by evaluating FIT performance among patients completing screening colonoscopy. We conducted our study among members of a nonprofit, group-model health maintenance organization (HMO) who were ages 50-75, at average risk for CRC, and due for CRC screening.

## Methods

### Summary

For this cohort study, we recruited patients referred for screening colonoscopy and requested that they complete two separate single-sample FITs labeled “1” and “2,” from two separate bowel movements. We then compared sensitivity and specificity estimates for 1-FIT and 2-FIT protocols among those who completed both FIT samples and the colonoscopy. Trained study staff analyzed the concentration of hemoglobin (ng/ml) for each FIT sample. Analysis occurred before colonoscopy completion; therefore, study staff was blind to colonoscopy results. We compared sensitivity and specificity estimates according to a range of hemoglobin concentration cutoffs for a positive test [21]:

- 50 ng/ml (10 μg/g)
- 75 ng/ml (15 μg/g)
- 100 ng/ml (20 μg/g)
- 125 ng/ml (25 μg/g)
- 150 ng/ml (30 μg/g)

### Study site

Kaiser Permanente Northwest (KPNW) is a nonprofit, group-model HMO with about 542,000 members in Southwest Washington and Northwest Oregon. It owns and operates two hospitals, contracts with six other local hospitals, and maintains 22 medical clinics (17 with primary care). Northwest Permanente, the medical provider group that serves KPNW patients, includes 797 physician members and 395 allied clinicians; of these, 265 are primary care providers. KPNW has a stable membership that is similar to the local insured community in terms of age, gender, race, and ethnicity. About 86% of members are white, about 7% are ethnically Hispanic, 6% are Asian, and 4% are African American. Membership turnover in the 50-plus age group averages 8.5% per year. Since 1996, KPNW has maintained complete electronic medical records (EMRs) for each health plan member, and its administrative and clinical databases are available for research purposes; members provide consent to use their data for research purposes upon enrollment or can opt out.

In 2009 KPNW updated its clinical-practice guideline pertaining to CRC screening. This guideline is based on recommendations of the US Preventive Services Task Force, which state that persons aged 50 to 75 and at average risk should be offered CRC screening. All of the United States Preventive Services Task Force (USPSTF)-recommended CRC-screening modalities are covered services.

### Subjects

We identified men and women aged 49-75 who received a referral for screening colonoscopy between December 1, 2011, and June 30, 2014, using the EMR. For each consecutive referred individual, we used three methods to apply exclusion criteria: 1) automated extraction from the medical record using diagnosis and referral codes, 2) chart review (of those with iron-deficiency anemia, to confirm no apparent known cause) and 3) detailed telephone interview to further screen and to determine interest. Table 1 lists the exclusion criteria. We excluded patients with dementia, end-stage renal disease, or HIV/AIDS to align the population with that of a randomized trial that utilized screening outreach exclusion criteria [22]. We developed symptom and anemia criteria by considering referral guidelines for suspected lower gastrointestinal cancer [23], as well as existing literature [24–26].

**Table 1:** Exclusion criteria for the study population

	Category of exclusion	Exclusion criteria
High risk diagnosis	History of cancer in the colon or rectum	Personal history of colorectal cancer
		Personal history of carcinoid of the colon
	Inflammatory bowel disease	Crohn’s disease
		Ulcerative colitis
	Inherited colorectal cancer syndrome	Familial adenomatous polyposis
		Peutz-Jegher’s syndrome
		Gardner syndrome
		Lynch syndrome
		Cowden syndrome
		Juvenile polyposis
		MYH-associated polyposis
	Unexplained iron-deficiency anemia	Men with a hemoglobin < 11, and women with a hemoglobin < 10, in combination with a ferritin of less than 100
		No reasonable explanation (e.g., recent surgery) for the anemia
	Recent weight loss	≥ 10% of body weight or ≥ 20 lbs. in the prior 6 months
	A combination of age and lower gastrointestinal symptoms suggestive of colorectal cancer	Age greater than 60 years, plus rectal bleeding for ≥ 3 months, and change in bowel habits toward looser stools or increased stool frequency persisting for six weeks or more
	History of adenomas	Prior history of adenomatous polyps
Recent endoscopy		Colonoscopy within 10 years
		Flexible sigmoidoscopy within 5 years
Excluded to align with the screening outreach population		Dementia
		End-stage renal disease
		HIV/AIDS
Colonoscopy not medically indicated		Currently receiving nursing home care
		Currently receiving hospice care
		Currently receiving active treatment for cancer
		Prior colectomy
Other exclusions		Needs an interpreter to communicate in English
		Opts out of research studies
		No available phone number

### Study materials

We mailed two FIT kits (OC-Auto FIT; Polymedco, Inc., Cortland Manor, NY) and instructions to participants who consented to be part of the study. No dietary or medication restrictions were advised. Participants were asked to label each sample with a date, and to collect the sample marked “1” on an earlier date, and sample “2” on a later date. Participants could use the enclosed pre-addressed, stamped envelope to return completed kits. We asked them to return both FIT kits within 7 days of completion of the initial FIT kit, to ensure that sample degradation would be minimal; we did not request that participants refrigerate the samples. When no date was recorded on the kit, we assumed that the kit marked “1” had been completed first and the kit marked “2” had been completed second. FIT kits were stored in a refrigerator for up to 2 weeks until they could be processed using the OC-Auto Micro 80 analyzer (Polymedco, Inc., Cortland Manor, NY).

### Reminders

Participants who did not complete the FIT kits or colonoscopy received up to three reminder calls and a reminder postcard.

### Colonoscopy

Patients who scheduled a colonoscopy within 180 days (6 months) and who completed the colonoscopy within 270 days (9 months) of completing FIT kits were included in the final cohort. Colonoscopies were conducted by 15 experienced staff gastroenterologists, each of whom had performed a minimum of 2000 colonoscopies previously. Gastroenterologists were blinded to the FIT results. One of three gastrointestinal pathologists (also blinded to FIT results) reviewed biopsy results. Participants received a standard bowel preparation including oral intake of 4 L of polyethylene glycol with electrolyte solution (Gavilyte) or sodium phosphate liquid (Fleet Phosphosoda). Gastroenterologists performed colonoscopies under conscious sedation using intravenous midazolam and fentanyl if desired.

### Histology

We reviewed colonoscopy results for each participant who completed a colonoscopy within 9 months of completing both FIT kits. Through manual abstraction of both colonoscopy report data and pathology data, we categorized the results of colonoscopy. Lesions were classified as non-neoplastic (e.g., hyperplastic), advanced adenomas, or low-risk adenomas. Advanced adenoma was defined as any adenoma ≥ 10 mm; or with tubular, tubulovillous, or villous histology or high-grade dysplasia (regardless of size); or ≥ 3 small (< 10 mm) tubular adenomas or serrated lesions [27, 28]. Low-risk (non-advanced) adenoma was defined as 1-2 tubular adenomas or serrated lesions < 10 mm in diameter, without high-grade dysplasia.

### Incidence of colorectal cancer within the screening age-eligible population

To contextualize the disease rates among individuals within the analytic cohort, we utilized the KPNW Certified Tumor Registry to assess the incidence of CRC among all health plan member adults ages 50-75 during the years 2009 and 2010. We computed incidence rates as cases occurring within 1 month of colonoscopy, using total person-years of observation of health plan members. The tumor registry database systematically captures site, histology, size, staging, and other information on all tumors identified among KPNW members since 1960, as mandated by the Commission on Cancer, Oregon and Washington State reporting guidelines, and agreements with the Center for Health Research. The registry maintains at least annual contact with clinicians and patients, and updates existing cases with survival data.

### Number of colonoscopy-detected colorectal cancers among the referral cohort

To further contextualize disease rates among individuals within the analytic cohort, we evaluated CRC incidence within the referral cohort that served as the initial recruitment source for participants in the study, utilizing electronic procedure codes and the Tumor Registry. Among those within the original referral cohort, but not recruited into the analytic cohort, we identified individuals who had completed a colonoscopy, then received a diagnosis of colorectal cancer within 1 month of completion. We performed chart review to confirm cases and assess whether each patient was of average or high risk for CRC prior to diagnosis.

### Analysis

We considered a positive result for either sample (at the specified cutoff) as an overall positive result within the 2-FIT cohort. We calculated sensitivity, specificity, negative predictive value, and positive predictive value for detection of advanced neoplasia for 1-FIT kit completion and 2-FIT kit completion at a range of hemoglobin concentration cutoffs—50 ng/ml (10 μg/g) to 150 ng/ml (30 μg/g). Differences between the AUCs (Area under the curve) for 1-FIT and 2-FIT generated by pre-specified cutoffs were tested using DeLong’s test for two correlated ROC curves [29], with the lowest hemoglobin concentration cutoff—50 ng/ml (10 μg/g) as the reference group. We also analyzed test performance using chi-square tests for logistic models by gender (male vs. female), by age range (50-64 vs. 65-75), and by a prior FIT having been completed in the health plan. We generated receiver-operating characteristic curves to evaluate effects of these variables on test performance of the 1-FIT kit, using methods as outlined by Gönen [30].

## Results

### Recruitment

**Fig. 1:**
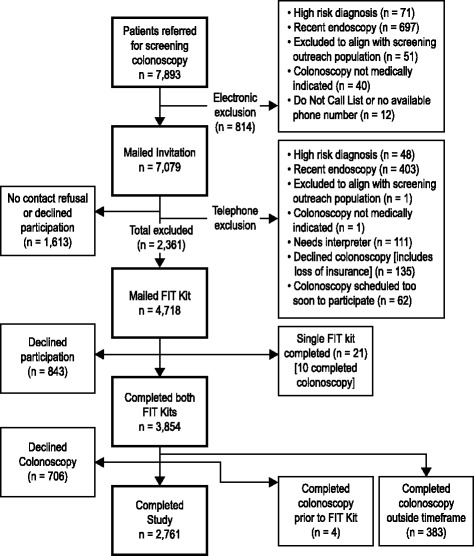
Study Flow Chart. Note that reasons for exclusion from being mailed an invitation (electronic exclusion) or from being mailed a FIT kit (telephone exclusion) occur at different stages and may overlap. The total ineligible at each step is provided. FIT = Fecal immunochemical test

Figure 1 shows patient recruitment, eligibility, and participation. We identified 7893 potential participants who had received a referral for screening colonoscopy. Among these, 2761 participants completed both FIT kits and a colonoscopy within 9 months of FIT kit completion. Mean time between dates written on the first and second FIT kits for those who completed both kits was 2 days. An additional 10 participants completed one FIT kit and a colonoscopy within 9 months of FIT kit completion.

### Demographics

Characteristics of the analytic cohort (*N* = 2771), in comparison to those who had received a referral for screening colonoscopy but did not end up in the analytic cohort (*N* = 5122), are shown in Table 2.

**Table 2:** Comparison of characteristics between analytic cohort and those referred but not in the analytic cohort

	Analytic Cohort		Not in Analytic Cohort		
	N	%	N	%	*p*-value
Gender					
Female	1422	51.32	2689	52.5	0.3158
Male	1349	48.68	2433	47.5	
Age at Referral					
50-64	2123	76.61	4086	79.77	0.0011
65-75	648	23.39	1036	20.23	
Previous FIT in EMR					
No	791	28.55	2450	47.83	<.0001
Yes	1980	71.45	2672	52.17	
All	2771	100	5122	100	

*p*-value from Chi Square test

Within the analytic cohort, 48.7% were male, and 89.1% were White; 3.7%, Asian; 2.2%, Black; 0.5%, Native American; and 2.4%, multiracial. Race was unknown for 53 participants (1.9%). Those who reported they were Hispanic totaled 113 (4.1%), and 95.9% were non-Hispanic. Also, 145 (5.2%) participants reported a family history of CRC in a first-degree relative, and 466 (16.8%) reported a family history of CRC in any relative.

### Colonoscopy results

Of all participants (*N* = 2771), the cecum was reached in 2688 (97%). Eighty-three (3%) of colonoscopies were considered incomplete; 55 (2%) of participants had insufficient bowel preparation. Table 3 shows colonoscopy findings. Within the analytic cohort, 779 participants (28.1%) had adenomas (advanced and non-advanced) and 209 (7.5%) had advanced adenomas. Sixty-two individuals (2.2%) had left-sided advanced neoplasia alone, and 150 individuals (5.4%) had isolated right-sided advanced neoplasia or a combination of right- and left-sided advanced neoplasia. Two participants (0.07%) had adenocarcinoma, and one had a carcinoid tumor. Of the two detected adenocarcinomas, one was Duke’s Stage A (T1N0M0), and the other was metastatic (T3N0M1b).

**Table 3:** Colonoscopy results based on most advanced lesion found

	Number	Percent
No polyps	1805	65.1%
Hyperplastic polyps	185	6.7%
Non-advanced adenoma	570	20.6%
Advanced adenoma	209	7.5%
3+ tubular adenomas or serrated lesions < 1 cm	92	3.3%
Tubular adenomas, serrated lesions > = 1 cm	43	1.5%
Polyps with high-grade dysplasia	74	2.7%
Colorectal adenocarcinoma	2	0.07%
Advanced neoplasia	211	7.6%
Total	2771	100%

### FIT sensitivity, specificity, positive predictive value and negative predictive value

**Fig. 2:**
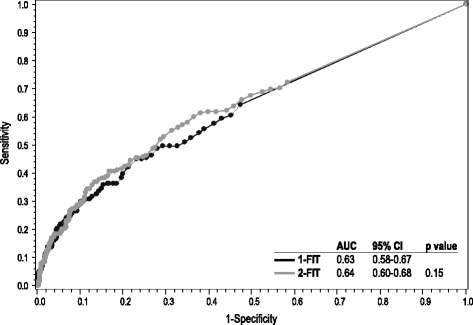
Receiver operating characteristic curves for advanced neoplasia, comparing 1-sample FIT and 2-sample FIT; demonstrates that there is no significant difference between ROC curves for 1-FIT and 2-FIT. Each line represents the highest fecal immunochemical hemoglobin measurement from the specified number of fecal samples collected and the corresponding maximum sensitivity and specificity at a given hemoglobin concentration cutoff, from 50 ng/ml (10 μg/g) to 150 ng/ml (30 μg/g). FIT-1 = first fecal immunochemical test sample collected; FIT-2 = both fecal immunochemical test samples collected; AUC = area under the curve

**Fig. 3:**
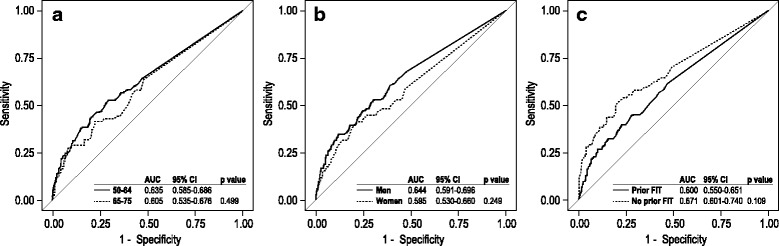
Receiver operating characteristic curves for advanced neoplasia with a 1-sample FIT, comparing different subgroups; demonstrates no difference in test performance of the 1-sample FIT by age group or gender, or by prior FIT completion. The figure shows ROC curves for a range of hemoglobin concentration cutoffs, from 50 ng/ml (10 μg/g) to 150 ng/ml (30 μg/g). **a** Comparing adults ages 50-64 to those ages 65-75. **b** Comparing men and women. **c** Comparing those who have completed a FIT to those who have not previously completed a FIT. FIT = Fecal immunochemical test; AUC = area under the curve

Table 4 shows the sensitivity, specificity, negative predictive value, and positive predictive value of both 1-FIT and 2-FIT protocols for detection of advanced neoplasia. Differences in sensitivity and specificity between the 1-FIT and 2-FIT protocols were not statistically significant (*p* > .05) at any of the pre-specified cutoff levels for the FITs. Figure 2 demonstrates that there is no significant difference between ROC curves for 1-FIT and 2-FIT. Table 5 shows comparisons of ROC curves for the 1-sample FIT at each of the hemoglobin concentration cutoffs, with 50 ng/ml (10 μg/g) as the reference group. There was a significant difference in test performance between 50 ng/ml (10 μg/g) and every other pre-specified comparison group (i.e., 75 ng/ml, 100 ng/ml, 125 ng/ml, and 150 ng/ml). Figure 3 demonstrates no difference in test performance of the 1-sample FIT by age group or gender, or by prior FIT completion.

**Table 4:** Performance of 1-sample and 2-sample FIT for advanced neoplasia at different hemoglobin concentration cutoffs

		% positive	Sensitivity			Specificity			PPV			NPV		
Test	Threshold (ng/ml)		Est.	LL	UL	Est.	LL	UL	Est.	LL	UL	Est.	LL	UL
1-FIT	50	7.58	22.6	17.0	28.3	93.7	92.7	94.6	22.9	17.2	28.5	93.6	92.7	94.5
2-FIT	50	11.99	29.7	23.6	35.9	89.5	88.3	90.7	19.0	14.8	23.2	93.9	92.9	94.8
1-FIT	75	5.09	16.5	11.5	21.5	95.9	95.1	96.6	24.8	17.7	32.0	93.3	92.3	94.2
2-FIT	75	8.09	23.1	17.4	28.8	93.1	92.2	94.1	21.9	16.5	27.3	93.6	92.7	94.6
1-FIT	100	4.19	14.2	9.5	18.8	96.6	95.9	97.3	25.9	17.9	33.8	93.2	92.2	94.1
2-FIT	100	6.50	19.3	14.0	24.7	94.6	93.7	95.5	22.8	16.7	28.9	93.4	92.4	94.4
1-FIT	125	3.61	13.7	9.1	18.3	97.2	96.6	97.9	29.0	20.1	37.9	93.2	92.2	94.1
2-FIT	125	5.42	18.4	13.2	23.6	95.7	94.9	96.5	26.0	19.0	33.0	93.4	92.5	94.4
1-FIT	150	3.03	12.3	7.9	16.7	97.7	97.2	98.3	31.0	21.1	40.8	93.1	92.1	94.0
2-FIT	150	4.66	17.5	12.3	22.6	96.4	95.7	97.1	28.7	20.9	36.5	93.4	92.4	94.3

**Table 5:** Test of differences between the Area Under the Curve (AUC) for pre-specified hemoglobin concentration cutoffs

	Reference group	Comparator group	Z - stats	*p*-value
Threshold (ng/ml)	50	75	2.3587	0.01834
AUC	0.58209	0.56223		
Threshold (ng/ml)	50	100	2.8421	0.00448
AUC	0.58209	0.55429		
Threshold (ng/ml)	50	125	2.7122	0.00668
AUC	0.58209	0.55485		
Threshold (ng/ml)	50	150	2.9666	0.00301
AUC	0.58209	0.55028		

*p*-value from DeLong’s test for two correlated ROC curves

### Incidence of colorectal cancer within age-eligible population

Among KPNW members ages 50-75 without a prior diagnosis of CRC, the unadjusted incidence rate of CRC in 2009-2010 was 1.08 per 1000 person-years (95% CI: 0.99-1.21).

### Colonoscopy-detected colorectal cancers within referral cohort

Of the 7893 individuals with a referral for screening colonoscopy, 5277 (66.9%) completed the colonoscopy within the study follow-up period. Nine of the 5277 received a diagnosis of colon or rectal cancer within 1 month of the colonoscopy (0.2%); of these, six were of average risk for CRC (two were in the analytic cohort; two had agreed to participate but did not follow through with FIT kit completion, two had declined participation), and three were not in the study because of high-risk features (i.e., suggestive bowel symptoms, inherited colorectal cancer syndrome).

## Discussion

Testing two fecal samples rather than one did not significantly improve FIT performance in detecting advanced neoplasia, at any pre-specified cutoff of hemoglobin concentration, among a younger, previously screened population in which advanced neoplasia incidence was low. Test performance did not vary by age category, gender, or prior FIT completion, indicating that targeting these subgroups would not significantly improve detection of advanced neoplasia within similar populations. A major limitation of this study was the low number of invasive cancers (2 out of 2,771), meaning that the findings pertain more specifically to test performance in detection of advanced adenomas.

Two prior studies of this FIT, with colonoscopy as the reference standard (one in a small, asymptomatic population and one in high-risk or symptomatic patients) had similar findings to those of this study; there was no significant difference in test performance between one, two, and three samples for detection of advanced neoplasia [31, 32]. An additional prior study of asymptomatic patients found that testing two samples with the OC-Micro FIT was more sensitive in detecting CRC than one sample [31]. This study was smaller, had a relatively high incidence of colorectal cancers [33], and drew its population mainly from specialty centers. Recent cohort studies comparing yield of CRC and advanced neoplasia between populations screening with 1-sample and 2-sample iterations (at the same hemoglobin concentration cutoff) of the same FIT used in this study found no significant difference in disease yield between groups [34], even after 2 rounds of screening [35]. Another cohort study found that the 2-sample iteration of this FIT yielded more cases with CRC and advanced neoplasia, but that comparable yield could be attained by lowering the hemoglobin concentration cutoff for the single-sample iteration of FIT [36].

There was an increase in sensitivity with a decrease in hemoglobin concentration cutoff, with significant differences in test performance when comparing 50 ng/ml (10 μg/g) to every successive higher preset hemoglobin concentration we studied (including the manufacturer recommended cutoff of 100 ng/ml [20 μg/g]). These findings confirmed trends noted in other similarly designed studies of the OC Auto FIT [31, 37, 38]. The optimal cutoff for a screening program will vary based on local resources. While lowering the hemoglobin concentration cutoff for FIT will make the test more sensitive for advanced neoplasia (in particular, proximal lesions) [39], there is a tradeoff in higher overall positivity rates and higher false positive rates [38]. Previously studied asymptomatic populations screened with the single-sample OC Auto FIT at the lower hemoglobin concentration cutoff of 50 ng/ml (10 μg/g) have had positive FIT results in 8-14% of participants on the first screening round [31, 35], although the proportion with a positive test result at this cutoff in our study was 7.6%. Maintenance of access to diagnostic colonoscopy for those with a positive FIT result remains an important consideration in implementing an organized CRC screening program based on mailed FIT kits [40–42], especially if the program also needs to maintain access to primary screening colonoscopy as an available option [43].

Notable strengths of our study include the prospective design, relatively large sample size, and thorough exclusion of high-risk individuals (including those with a prior history of adenomas and those with symptoms suggestive of colorectal cancer). This is the largest prospective cohort study evaluating test performance of different iterations of this quantitative FIT, and colonoscopy served as the reference standard for all participants. The study occurred in a real-world integrated care setting, where participants had access to CRC screening (and the majority had previously completed screening). Our study also had limitations. The number of participants with advanced neoplasms was low, and so we do not address the question of whether testing of two samples would detect more colorectal cancers; we did not have enough cancer cases to assess this endpoint. Our one-time sensitivity estimates for advanced neoplasia are lower than those in prior studies with a higher incidence of colorectal neoplasms, due to spectrum effect [33, 44–46]; this makes estimation of ‘true’ sensitivity for this FIT challenging. Lastly, because of fluctuation in and varying durations of health plan membership, we were unable to accurately assess the proportion of patients who had had a prior negative endoscopy (e.g., a colonoscopy greater than 10 years prior or a flexible sigmoidoscopy greater than 5 years prior to enrollment).

## Conclusions

Our study findings confirm that a FIT outreach program within the United States, where overall screening rates in the country approximate 60% [47], would most efficiently utilize a single-sample FIT. Decreasing the hemoglobin concentration cutoff from the manufacturer-recommended cutoff of 100 ng/ml (20 μg/g) to 50 ng/ml (10 μg/g) significantly increased sensitivity of the single-sample FIT for advanced neoplasia. Further research should continue to study initiation of organized screening programs to replace opportunistic screening, especially within health systems in which screening is a covered benefit [48]. Further research should also optimize the reach [49] of organized screening programs across different settings that have variable access to diagnostic colonoscopy [50].

## Abbreviations

AUC: Area under the curve
CRC: Colorectal cancer
EMR: Electronic medical record
FIT: Fecal immunochemical test
HMO: Health maintenance organization
KPNW: Kaiser Permanente Northwest
ROC: Receiver-operating characteristic curves
USPSTF: United States Preventive Services Task Force
